# Grapevine Green Pruning Residues as a Promising and Sustainable Source of Bioactive Phenolic Compounds

**DOI:** 10.3390/molecules25030464

**Published:** 2020-01-22

**Authors:** Stefano Acquadro, Silvia Appleton, Arianna Marengo, Carlo Bicchi, Barbara Sgorbini, Manuela Mandrone, Francesco Gai, Pier Giorgio Peiretti, Cecilia Cagliero, Patrizia Rubiolo

**Affiliations:** 1 Dipartimento di Scienza e Tecnologia del Farmaco, Università di Torino, Via Pietro Giuria 9, 10125 Torino, Italy; stefano.acquadro@unito.it (S.A.); silvia.appleton@edu.unito.it (S.A.); arianna.marengo@unito.it (A.M.); carlo.bicchi@unito.it (C.B.); barbara.sgorbini@unito.it (B.S.); cecilia.cagliero@unito.it (C.C.); 2 Dipartimento di Farmacia e Biotecnologie, Alma Mater Studiorum—Università di Bologna, Via Irnerio 42, 40126 Bologna, Italy; manuela.mandrone2@unibo.it; 3 Istituto di Scienze e delle Produzioni Alimentari, National Research Council, L.go Paolo Braccini 2, 10095 Grugliasco (TO), Italy; francesco.gai@ispa.cnr.it (F.G.); piergiorgio.peiretti@ispa.cnr.it (P.G.P.)

**Keywords:** *Vitis vinifera* L., green pruning residues, viticulture sustainability, phenolic pattern, antioxidant activity, HPLC-PDA-ESI-MS/MS, colorimetric in vitro assays

## Abstract

Green pruning residues (GPRs) and leaves from 16 red and white *Vitis vinifera* L. cultivars from Piedmont (Italy) were studied. The investigated samples were extracted by ultrasound-assisted extraction optimized by an experimental design, and quali- and quantitatively analyzed by HPLC-PDA-MS/MS. GPRs and leaves show a similar polyphenolic pattern, with quercetin 3-*O*-glucuronide, caftaric acid, and quercetin 3-*O*-glucoside as the main components, although in variable proportions. The HPLC results were related to the antioxidant activity, measured as total phenolic content and through DPPH and ABTS assays with similar results. Colorimetric in vitro assays, offline combined with HPLC-PDA analysis, determine which compounds contribute to the antioxidant activity in terms of radical scavenging abilities. Valorization of GPRs is a potential source of natural compounds that could be of interest in the health field, increasing their economic value together with a positive effect on the environment.

## 1. Introduction

Wine production and, thereby, grape crops are one of today’s main and most widespread agro-economic activities, with more than seven million hectares cultivated and 77 million tons produced worldwide in 2016 [1]. Unfortunately, viticulture produces huge amounts of residues, which are a serious economic issue; they are mainly destined to composting or discarded in open areas, potentially causing environmental problems [2]. Solutions involving reuse, recycling, and recovery of resources should, therefore, be found to reduce the amount of waste. The “waste” issue related to environmental sustainability has recently been the object of attention from several regulatory institutions (e.g., Directives 1999/31/EC and 2008/98/EC from the European Commission). The exploitation of by-products generated by grape crops is, therefore, currently of great interest to reduce the environmental impact of wine production, as well as meet the growing demand for green materials and renewable sources of nutrients and bioactive compounds for the feed, functional food, and food supplement industries [3,4,5,6,7].

In the Mediterranean area, and in Italy in particular, wine production and vineyard cultivation are widespread, and result in large amounts of pruning residues waste. In 2011, 3218 million hectares were cultivated as vineyards in Europe [8] of which 736 million hectares were in Italy, for a production of 7487 million tons of grapes [9]. Referring to European data, potential residues derived from grapevine cultivation are estimated to be about 1.4 million tons of dry matter (DM) [10]. Sánchez et al. [11] estimated the amount of vineyard pruning residues in a range of 1 to 7.5 t/ha. The most vigorous pruning is in late summer, but the selective removal of grapevine leaves (together with fruitless young twigs) during spring also generates large amounts of interesting by-products. This operation is carried out to ventilate and improve the lighting of grape bunches during ripening and consists of removing some or all of the leaves present at the level of the basal area of the shoots, where a lot of bunches are present. The removal process results in a noticeable increase in the content of anthocyanins and other flavonoids [12]. These by-products are known as “green pruning residues” (GPRs) [13].

Many grapevine by-products, such as grape pomace, seeds, and stems, have been characterized in depth, in terms of both chemical composition and biological properties [14,15,16,17,18,19,20,21,22]. The same is true for vine leaves; these by-products are traditionally used for human and animal consumption, as food [23], animal feed [24], ingredients of dietary supplements [25], and in cosmetics [26].

As for other parts of the grapevine, leaves are mainly characterized by phenolic compounds as specialized metabolites; in particular, phenolic acids, flavonols (mainly in the form of *O*-glycosides of quercetin and kaempferol) and, to a lesser extent, by stilbenes (resveratrol), flavan-3-ols, and anthocyanins (mainly in red autumn leaves) [7,23,27,28]. The beneficial properties of grapevine leaves are attributed to these phenolic compounds and are principally correlated to the well-known antioxidant activity [7,17,25,29,30,31].

One of the main limitations to re-using the leaves as by-products is that they are harvested in late summer, after potential treatment(s) with pesticides. Such treatments are a matter of concern for animal feed, because they could induce toxic effects [32]. Conversely, GPRs are harvested during the spring before any treatment has been applied, and therefore are free of pesticide residues, and therefore can be considered as potential health by-products to be exploited. However, to the best of the authors’ knowledge, no information concerning the phytochemical phenolic composition or antioxidant properties of these by-products is available. GPRs should not be confused with the “vine pruning residues” that are collected during winter, and therefore are a woody material with a completely different composition (i.e., cellulose, lignin, and other phenolic compounds) [4,33].

Taking into account the above considerations, this study aims to investigate the potential of grapevine green pruning residues (GPRs) as a source of antioxidants, by evaluating their phenolic composition and comparing the results with those of the already exploited late-summer leaves. These investigations involved several cultivars of *V. vinifera* L., both red and white, harvested in Piedmont (Italy) and used to produce some of the most prestigious wines. The phenolic composition of GPRs and leaves was determined through HPLC-PDA-ESI-MS/MS analysis after their extraction with an ultrasound-assisted extraction (UAE) method, previously optimized through an experimental design approach [34]. The antioxidant properties of the extracts were investigated through colorimetric invitro assays (scavenging of the 2,2-diphenyl-1-picrylhydrazyl (DPPH^•^) and the 2,2’-azinobis-3-ethyl-benzthiazoline-6-sulphonate (ABTS^+•^) radicals) and the results were correlated to those obtained by HPLC and total phenol content assay. The antioxidant properties of the matrices were also confirmed by combining the colorimetric in vitro assays offline with HPLC-PDA analysis, to determine which compounds contribute most to the antioxidant activity in terms of radical scavenging abilities [35].

## 2. Results and Discussion

### 2.1. Phytochemical Analysis of Grapevine Green Pruning Residues (GPRs)

Grapevine phytochemical composition was studied by HPLC-PDA-MS/MS. The analyses were performed on both green pruning residues and leaves on samples from the same plants, because only information for the leaves is available from the literature [7,23,27,28]. The results were then compared to evaluate differences and similarities in the chemical composition of the two investigated samples.

#### 2.1.1. Optimization of the Extraction of the Phenolic Compounds of GPRs

The GPRs were extracted by ultrasound-assisted extraction (UAE), an easy-to-handle, technique that can be run at room temperature, providing correct extraction of thermolabile compounds. Moreover, it is a green chemistry technique, entailing low solvent and energy consumption while providing a high extraction yield and fast kinetics [36,37].

The extraction method was optimized through experimental design, i.e., an approach giving the most effective combination of parameters to run the ultrasound extraction process to obtain the highest polyphenolic yield. A pool sample of GPRs, which was obtained by mixing an equal amount of GPRs from each cultivar, was used as the model sample for optimization.

The first step was to select the optimal extraction solvent. Ethanol, acetone, and a mixture of methanol/water (70:30 *v*/*v*) were chosen for the GPRs pool sample extractions, based on the literature [7]. HPLC analysis of the different extracts revealed that the three solvents gave comparable qualitative results, but methanol/water provided the highest recovery (data not shown). 

The main variables affecting extraction (i.e., solvent, extraction time, amount of matrix, and volume of solvent) were then screened by applying a Box, Hunter and Hunter design (see Table S1). For temperature, it is important to consider that higher temperatures can improve mass transfer during extraction, but, at the same time, it can promote a high component degradation rate, in particular above 75 °C [38]. The UAE process was, therefore, carried out at 30 °C.

The extraction efficiency of each experiment was evaluated in terms of both the UV peak area of every compound detected at 270 nm, and of the sum of the areas of all peaks. 

The effects of the variables on the sum of all peak areas, and the peak area of the most representative compounds, are illustrated by Pareto charts in Figures S1 and S2 (a1–a4), respectively. All variables were significant (*p* < 0.05) with an increase of the response when passing from the lowest to the highest level. 

The final optimization of the variables most influencing the extraction process (i.e., volume of solvent and amount of matrix) was carried out applying a central composite design (CCD) keeping extraction time (15 min) and water concentration (30%) in the water/methanol solvent mixture constant (Table S1).

Surface response plots show the relationships between extraction parameters and analyte response (extraction yield). Figures S1B and S2 (b1–b4) show the response surface plots correlating the effect of matrix amount and solvent volume to both the extraction yield of the most abundant phenols and the sum of all peaks. All surface responses clearly indicate that extraction yield improves as the two variables increase. The mathematical model developed showed good consistency between experimental and predicted values (data not shown).

In conclusion, the optimum extraction conditions were the following: 15 min extraction time, 30% water in the solvent mixture, 500 mg matrix, and 50 mL solvent.

To make the method quicker and more sustainable, extraction was carried out reducing both the amount of plant material and the solvent volume, while maintaining the ratio between them constant. Under the new conditions (i.e., 15 min extraction time, 30% water in the solvent mixture, 100 mg matrix, and 10 mL solvent), the extraction results were perfectly comparable with the previous results.

Moreover, the single-step process was also exhaustive as it was confirmed by the lack of peaks detected in the chromatogram of a second extraction on the same plant material (data not shown).

#### 2.1.2. Identification and Quantification of the Phenolic Compounds of GPRs

**Figure 1:**
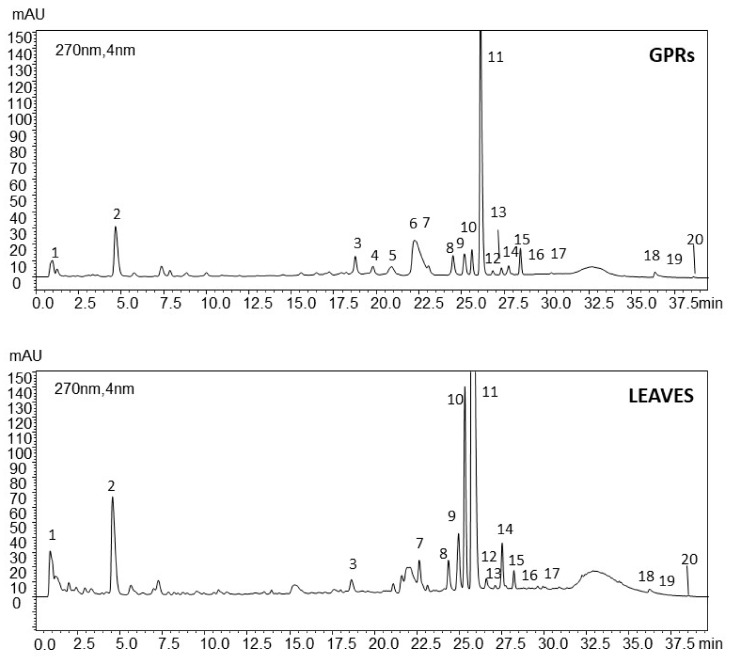
HPLC-PDA profiles (λ, 270 nm) of green pruning residues (GPRs) and leaves. For peak numbers see Table 1.

To the best of the authors’ knowledge, no data are available on the phenolic composition of *V. vinifera* green pruning residues (GPRs), whereas some phytochemical investigations on grapevine leaves have been reported [7,23,27,28]. Therefore, these studies were taken as a reference, since HPLC-PDA-MS/MS patterns of GPRs and leaf hydroalcoholic extracts showed similar chemical compositions (Figure 1), with a marked abundance of phenolic compounds. 

Chromatographic patterns, UV, and mass spectral data detected 20 informative compounds. The UV spectra for each peak provided a preliminary indication of the group of compounds. The molecular weight of each peak was also defined by its mass spectral pattern, through the complementary correspondence between positive and negative pseudomolecular ions in ESI^+^ and ESI^−^ modes. The product ion scan analysis of the pseudomolecular ions under investigation provided diagnostic fragments for each compound. The identity of 11 compounds in the extract was confirmed by co-injection of authentic commercial standards, whereas eight peaks were putatively identified by comparison with literature data (Table 1).

In particular, compounds for which authentic commercial reference standards were not available were tentatively identified through their tandem mass spectrometry fragmentation pattern, as reported by Marengo et al. [44]. This approach provides further structural information on unknown compounds. For example, vitilagin (or isovitilagin) was tentatively identified by its UV spectral data (UV_max_ = 275 nm), pseudomolecular ions 803 m/z and 801 m/z, in ESI^+^ and ESI^−^ ionization modes, respectively, and fragmented to give diagnostic ions at m/z 153, 337, and 633 in ESI^+^ and m/z 765 in ESI^−^ [43]. Other hydrolyzable tannins were putatively hypothesized on the basis of their UV maximum spectral absorption and diagnostic fragments at m/z 753, 301, 273, and 229 [39,41,42]. Selected reaction monitoring acquisition (SRM) was also carried out on specific product ions, providing further structural information on the investigated compounds. 

In agreement with data reported for polyphenols of *V. vinifera* leaves, flavonoids were the most representative group in GPRs hydroalcoholic extracts, in particular in the form of *O*-glycosides of quercetin, kaempferol, myricetin, and rhamnetin (or isorhamnetin). A phenolic acid derivative (i.e., caftaric acid) and some hydrolyzable tannins (including vitilagin and isovitilagin) were also detected [23,40,43]. Interestingly, GPRs extracts did not contain stilbenes, although traces of resveratrol were detected in leaves [28].

**Figure 2:**
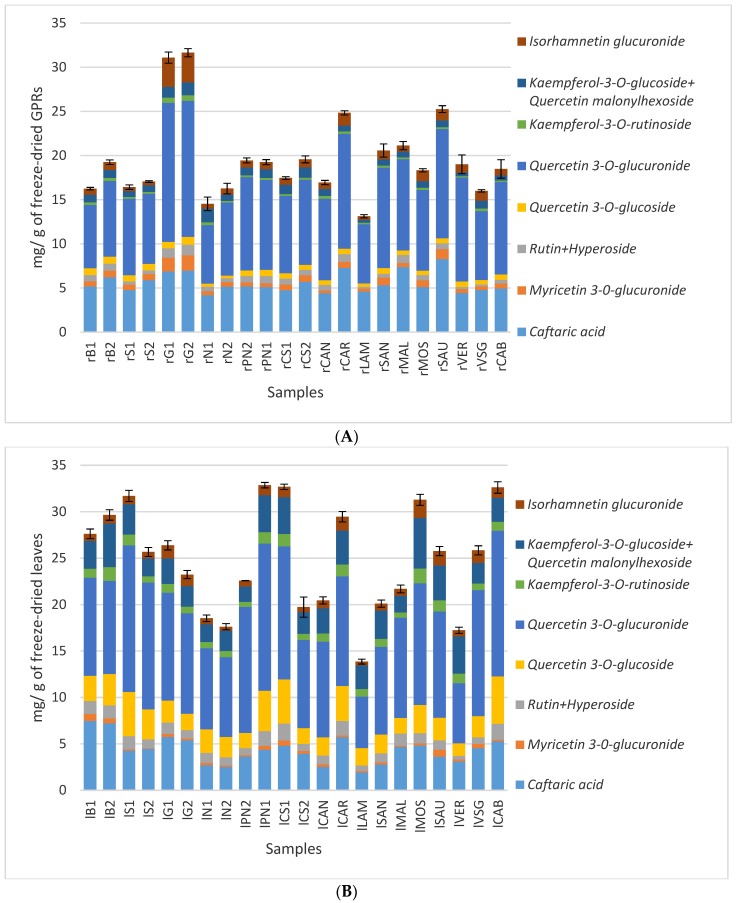
Concentration (mg/g) of the main phenolic compounds of freeze-dried green pruning residues (GPRs) (**A**) and leaves (**B**).

Figure 2 shows the quantitative results on GPRs and leaves highlighting the quantity of each target compound in relation to the total amount of phenolic compounds quantified. It is noticeable that the total content of phenolics, as well as their proportions, are similar in GPRs and in leaves, quercetin 3-*O*-glucuronide, caftaric acid, and quercetin 3-*O*-glucoside being the most abundant compounds in both matrixes. The total phenolic content determined by the Folin–Ciocalteu method is in accordance with HPLC quantification. The concentration of total phenolics was expressed as mg of gallic acid equivalent (GAE) per g of sample and no statistical differences emerged among GPRs and leaves (Figure S3).

#### 2.1.3. Statistical Analysis and Comparison of the Phytochemical Patterns of GPRs and Leaves

Since *V. vinifera* GPRs and leaves showed very similar polyphenolic profiles, principal component analysis (PCA), an unsupervised multivariate data analysis method, was applied to evaluate the possibility of discriminating between the two matrices, using as variables, the eight components quantified.

**Figure 3:**
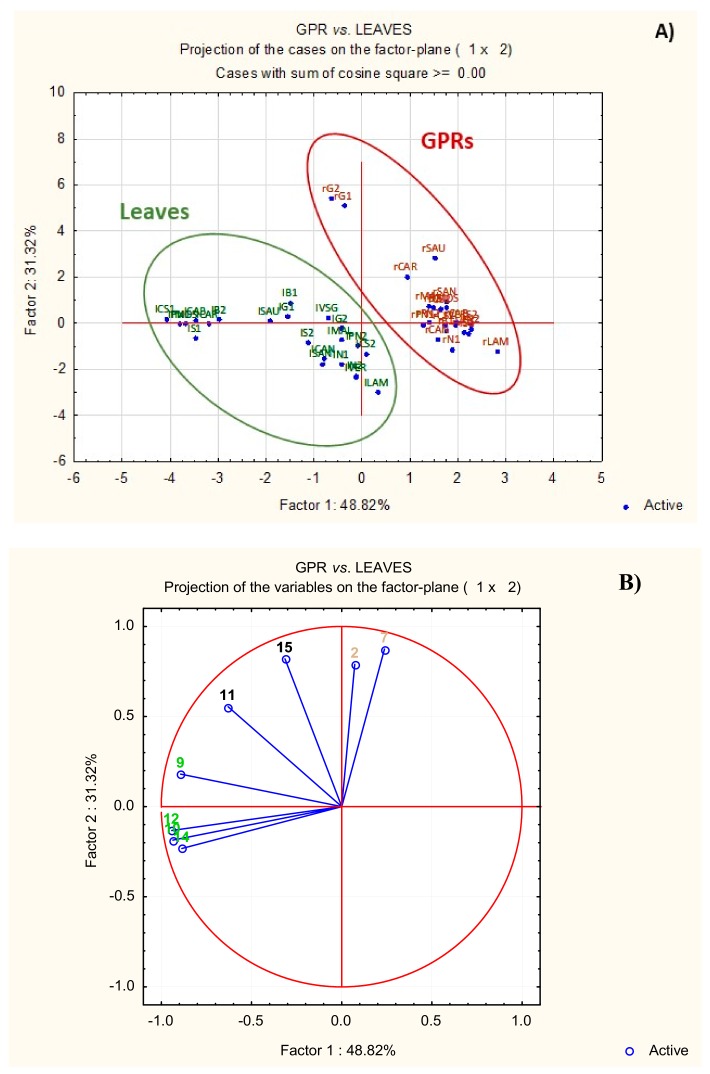
Score plot (**A**) and loading plot (**B**) of the principal component analysis relative to the quantity of the main phenolic compounds in green pruning residues (GPRs) (r) and leaves (l). In the score plot, GPRs are in brown and leaves in green. In the loading plot: (2), caftaric acid; (7), myricetin glucuronide; (9), rutin and hyperoside; (10), quercetin 3-*O*-glucoside; (11), quercetin 3-*O*-glucuronide; (12), kaempferol-3-*O*-rutinoside; (14), kaempferol-3-*O*-glucoside and quercetin malonylhexoside; and (15), isorhamnetin glucuronide. The analytes significantly more abundant in GPRs are in brown and those significantly more abundant in leaves are in green.

The scores and loading plots are shown in Figure 3. The first (Factor 1) and second (Factor 2) components (48.82% and 31.32% of explained variation, respectively) clearly discriminate between GPR and leaf samples. A Student t-test was, therefore, applied to each variable; Figure S4 shows the box plots for the compounds quantified, to compare their abundance in GPRs and leaf samples. The box plots show that caftaric acid and myricetin glucuronide are more abundant in GPR extracts (*p* < 0.01) and positively correlated with the second principal component (Figure 3B). Conversely, rutin and hyperoside, quercetin 3-*O*-glucoside, kaempferol-3-*O*-rutinoside, and kaempferol-3-*O*-glucoside and quercetin malonyl hexoside are more abundant in leaf extracts (*p* < 0.01) and negatively correlated with the first principal component (Figure 3B). The amounts of quercetin 3-*O*-glucuronide and isorhamnetin glucuronide in GPRs and leaves (*p* > 0.01) were not significantly different.

These results indicate that, although *V. vinifera* GPRs and leaves have similar chemical patterns, there are some differences in the relative abundances of common components.

### 2.2. Evaluation of the Antioxidant Potential of GPRs 

#### 2.2.1. In Vitro Antioxidant Assays (Scavenging of DPPH^•^ and ABTS^+•^ Radicals)

In a second step, the antioxidant properties of the extracts were investigated through colorimetric in vitro assays and the results were correlated to those obtained by HPLC. In particular, the antiradical activity was measured by evaluating the scavenging effects on the 2,2-diphenyl-1-picrylhydrazyl (DPPH^•^) and the 2,2’-azinobis-3-ethyl-benzthiazoline-6-sulphonate (ABTS^+•^) radicals.

The scavenging effect on ABTS^+•^ radicals was expressed in terms of Trolox equivalent antioxidant capacity (TEAC), and the scavenging effect on DPPH^•^ radicals as EC50. 

**Figure 4:**
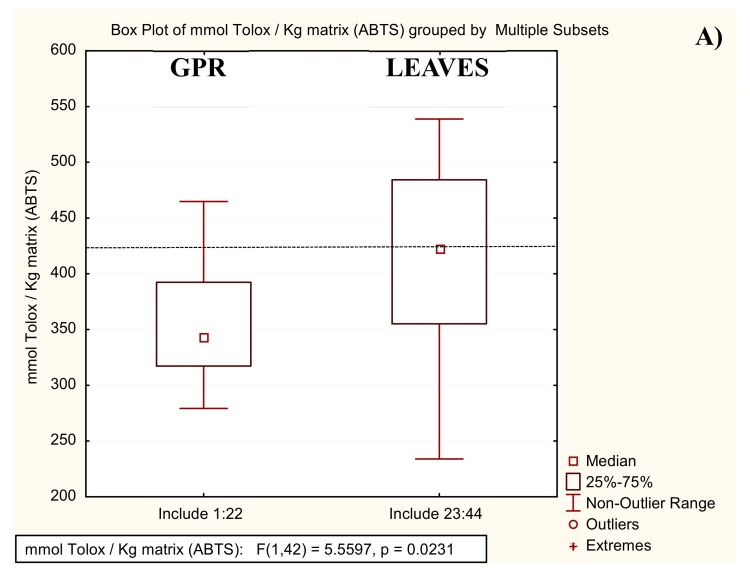

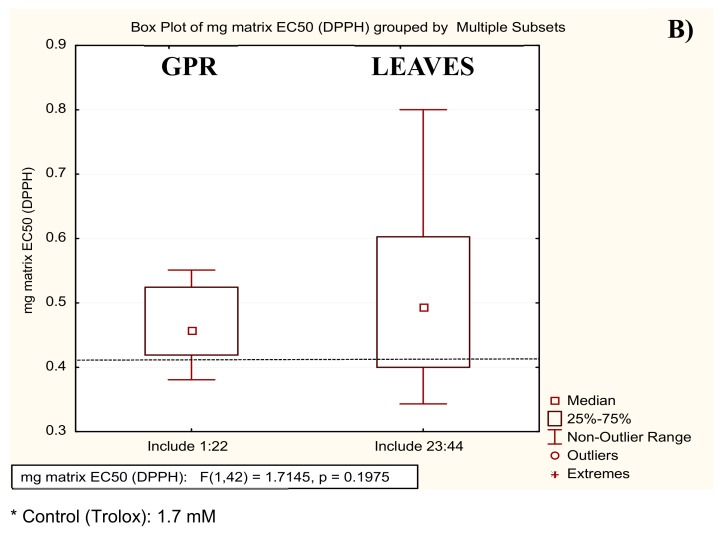
Box plots relative to the in vitro colorimetric antioxidant assays: Trolox equivalent antioxidant capacity (TEAC) by ABTS Assay expressed as mmol Trolox/kg matrix (**A**) and EC50 (mg matrix) by DPPH assay (**B**). The dashed line gives the literature values (i.e., [30] for **A**, and [31] for **B**).

The results are summarized in Figure 4 and Figure S5. The leaves’ antioxidant activity is in close agreement with reported data [30] and no significant differences emerged between GPRs and leaves (*p* > 0.01 for all antioxidant assays, Figure 4), although all assays showed that the leaf extracts appear more variable than that of the GPR samples. These results indicate that GPRs can be assumed to be a source of antioxidant compounds equivalent to already exploited grapevine leaves.

The possible correlation between the two antioxidant assays was also investigated and compared with HPLC quantitation data and the total phenols assay. Table S2 shows the Pearson correlation coefficients between the different measurements. The results with the four test techniques are consistent; their correlation is statistically different from zero, with a significance level α = 0.05 with HPLC quantitative data, total phenolic content and TEAC correlated positively, and EC50 values on DPPH^•^ correlated negatively to the other results.

#### 2.2.2. Offline Combination of Antioxidant Assays with HPLC-PDA Analysis

The objective of this part of the study was to establish an efficient method for quickly identifying antioxidant active components in the phenolic extracts. Therefore, a further investigation was run to screen the component(s) of the extract(s) that mainly contributed to the antioxidant properties, in terms of their radical scavenging ability [35]. This information was obtained by analyzing with the HPLC-PDA system, the samples preliminarily submitted to the in vitro radical treatment. The HPLC profiles of GPR and leaf extracts, before and after reaction with DPPH^•^ and ABTS^+•^ radicals, were compared to determine the radical-scavenging activities of each phenolic component, via the percent reduction of their peak areas. 

**Figure 5:**
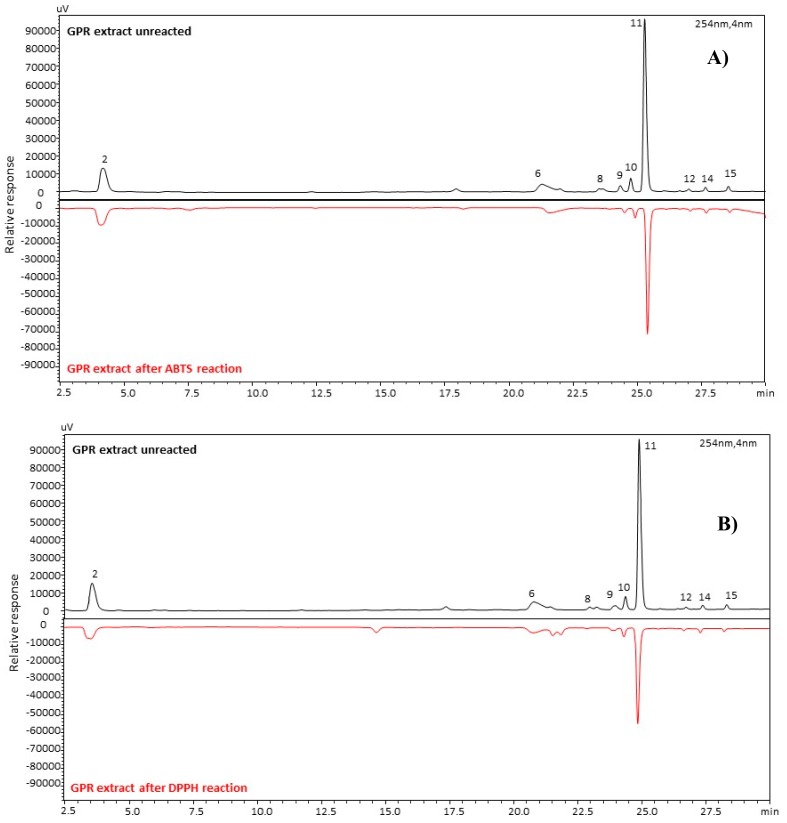
HPLC chromatograms of GPRs extracts before (black profile) and after (red profile) reaction with ABTS (**A**) and DPPH (**B**) free radical. For peak numbers, see Table 1.

Figure 5 shows the comparison of the GPRs profiles, before and after reaction with ABTS^+•^ (Figure 5A) and DPPH^•^ (Figure 5B), and percent peak area reduction of the analytes of GPRs and leaves, after reaction with the two radicals, are listed in Table 2.

As expected, the results obtained with the two groups of samples were consistent, while some differences in scavenging activities were found between compounds and within the two radicals. In particular, compound **8** shows a very high scavenging activity (with about 70% reduction) with both DPPH^•^ and ABTS^+•^. The % reductions of caftaric acid and tannin **6** for both assays were around 30%. Conversely, the different scavenging activity of the flavonoids is noteworthy, in particular against DPPH^•^. All quercetin glycosides show a percent reduction around 30% with both DPPH^•^ and ABTS^+•^ radical, while kaempferol and rhamnetin glycosides show lower reduction with ABTS^+•^ and lower again with DPPH^•^. This is probably because quercetin has an additional free hydroxyl group as compared with the other aglycones. These results are in agreement with those of Zhao et al. [35].

## 3. Materials and Methods 

### 3.1. Plant Material and Growth Conditions

The plant material was harvested during the 2016 and 2017 growing seasons, in an experimental vineyard located in Piedmont (North West Italy). The climate at this site (293 m a.s.l., 45°03′58″N/7°35′37″E) is temperate subcontinental, characterized by two main rainy periods, in spring and autumn. During the growing season, total precipitation ranges from 139 mm/month (July) to 76 mm/month (May), and the mean temperature and mean relative humidity are 20.3 °C and 68.6%, respectively. Conventional agronomic management was regularly applied in the vineyard, cultivated in soil taxonomically classified as entisol according to the USDA soil classification system [45] which is sandy soil, low in organic matter, with a moderately alkaline pH. 

Samples of GPRs and leaves of eleven varieties of red grapevine (*Vitis vinifera* Cvs.: Barbera, Cabernet Franc, Cabernet Sauvignon, Canaiolo Nero, Carignano, Grenache, Lambrusco Salamino, Nebbiolo, Pinot Noir, Sangiovese, and Syrah) and five varieties of white grapevine (*Vitis vinifera* Cvs.: Malvasia Bianca, Moscato Bianco, Sauvignon Blanc, Verdicchio, and Vernaccia) were collected in duplicate from standard vertical trellises, with edging shears (see Table S3). Sampling was done in the morning after dew evaporation, during June for GPRs and September for leaves, and was never carried out on rainy days. Fresh GPRs and leaf samples were immediately frozen and freeze-dried using a lyophilizer (5 Pascal, Trezzano sul Naviglio, Italy), and then ground in a Cyclotec mill (Tecator, Herndon, VA, USA) to pass through a 1 mm screen, and stored.

### 3.2. Chemicals

HPLC-grade acetonitrile (LC-MS grade), methanol, ethanol, petroleum ether, formic acid (>98% purity), Folin–Ciocalteu’s phenol reagent, 1,1-diphenyl-2-picrylhydrazyl radical (DPPH^•^), 2,2′-azino-bis(3-ethylbenzothiazoline-6-sulfonic acid) diammonium salt (ABTS^TM^), potassium persulphate, (±)-6-hydroxy-2,5,7,8-tetramethylchromane-2-carboxylic acid (Trolox), gallic acid, quercetin, kaempferol, resveratrol, and rutin were supplied by Merck (Milan, Italy). De-ionized water (18.2 MΩ cm) was obtained from a Milli-Q purification system (Millipore, Bedford, MA, USA). Quercetin 3-*O*-glucoside, kaempferol-3-*O*-rutinoside, kaempferol-3-*O*-glucoside, and hyperoside were supplied by Extrasynthese (Genay Cedex, France). Rhamnetin, isorhamnetin, caftaric acid, and quercetin 3-*O*-glucuronide were from Phytolab (Vestenbergsgreuth, Germany).

### 3.3. Extraction Method

Extraction conditions (amount of plant material, type of solvent, solvent volume, and extraction time) were carefully optimized through experimental design (see Section 2.1.1). A pool sample of GPRs obtained by mixing an equal amount of all cultivars was prepared to run the experimental design experiments. The optimized extraction conditions were as follows: First, 0.100 g of each sample was extracted with an ultrasonic bath (Soltec, Sonica S3 EP 2400) operating at 40 KHz with 10 mL of methanol/water (70:30, *v*/*v*) for 15 min. The ultrasonic bath temperature was set at 30 °C and checked before and after each extraction. The supernatant was centrifuged at 4500 rpm for 10 min and poured into a separatory funnel together with 5 mL of petroleum ether, to reduce chlorophyll interference, by liquid–liquid extraction. The aqueous layer was evaporated in a rotary evaporator under vacuum to a volume of about 1 mL, at a temperature below 50 °C in order to avoid phenol degradation. The extract was then diluted to 2 mL with methanol and filtered through a PTFE 0.22 μm syringe hydrophilic filter for LC analysis. Extraction was repeated thrice for each sample.

### 3.4. HPLC-PDA-MS/MS Analysis and Quantification

#### 3.4.1. Qualitative Analysis

A Shimadzu Nexera ×2 system was used for qualitative analysis; it was equipped with a photodiode array detector SPD-M20A in series to a Shimadzu LCMS-8040 triple quadrupole system with an electrospray ionization (ESI) source (Shimadzu, Dusseldorf Germany). 

An Ascentis Express RP-Amide column (10 cm × 2.1 mm, 2.7 μm, Supelco, Bellefonte, USA) was used; mobile phase A was water/formic acid (999:1, v/v) and mobile phases B was acetonitrile/formic acid (999:1, v/v), respectively. The flow rate was 0.4 mL/min, and the column temperature was 30 °C. The gradient program was as follows: 0 to 3 min 5% B, 3 to 20 min 5% to 15% B, 20 to 30 min 15% to 25% B, 30 to 42 min 25% to 75% B, 42 to 52 min 75% to 100% B, and 52 to 53 min 100% B. The total analysis time including pre- and post-running was 60 min. UV spectra were acquired from 220 to 450 nm.

Mass spectrometer operative conditions were as follows: Heat block temperature, 200 °C; desolvation line (DL) temperature, 230 °C; nebulizer gas (N_2_) flow rate, 3 L/min; and drying gas (N_2_) flow rate, 15 L/min. Full scan mass spectra were acquired from 50 to 2000 m/z both in positive and in negative scan mode, with an event time of 0.5 s.

When pseudomolecular ions [M + H]^+^ in ESI^+^ or [M − H]^−^ in ESI^−^ were identified, they were submitted to collision (collision energy, -35.0 V for ESI^+^ and 35.0 V for ESI^−^) in product ion scan mode with an event time of 0.2 s. Selected reaction monitoring acquisition on specific product ions derived from precursor ion fragmentation was performed. Retention times, UV, and MS spectra were used to identify and tentatively identify the main components of the extracts. These data were compared with those of authentic commercial standards or when not available, to the literature data (see Table 1 and Section 2.1.2).

#### 3.4.2. Quantitative Analysis 

Each extract (5 μL) was analyzed in triplicate with a Shimadzu UFLC XR (Shimadzu, Dusseldorf, Germany) equipped with a photodiode array detector SPD-M20A using the same column, mobile phases, flow rate, and gradient program as in the qualitative analysis.

UV spectra were acquired in the 220–450 nm wavelength range, and the resulting chromatograms were integrated at 270 nm, to process the analysis for the offline combination of antioxidant assays and LC analysis, and at the λ_max_ of the identified peaks (see Table S2) for quantitative analysis.

Quantitation was performed by an external standard calibration method, and the results expressed as mg of compound per g of matrix (mg/g). The calibration curves of caftaric acid and quercetin 3-*O*-glucuronide were built up by analyzing them at five concentrations in methanol/water (30:70 *v*/*v*) in the range 100–1000 mg/L, while for rutin, quercetin 3-*O*-glucoside, and kaempferol 3-*O*-glucoside, seven concentrations in the range 5–250 mg/L were used. Quantitation was performed on eight target compounds, using the calibration curves built up on the same compound or, when not available, with those of compounds belonging to the same chemical class. The calibration curves and the analytical performances of the method are in Table S4. The analytical performances were measured in terms of repeatability (RSD% never exceeding 5%) and intermediate precision (RSD% never exceeding 10%).

All data were processed using LabSolution software (Shimadzu, Dusseldorf Germany).

### 3.5. Total Phenolic Content Assay

The total phenolic content was determined as described by Singleton and Rossi [46] with slight modifications. First, 250 µL of the extracts (diluted 1:25 in methanol) were added to 4 mL of water, together with 250 µL of pure Folin–Ciocalteu’s phenol reagent, and 500 µL of an aqueous solution of Na_2_C0_3_ (pH = 10). Then, the absorbance was measured at 765 nm after one hour with a UV/Visible spectrophotometer (Genesys 6, Thermo Electron Co., Madison, WI, USA). The results were expressed as mg of gallic acid equivalent (GAE) per g of matrix. The calibration curve of gallic acid was built up with the same method, by analyzing its standard at concentrations ranging from 0.01–0.5 mg/mL.

### 3.6. Antioxidant Activity Determination

#### 3.6.1. Scavenging Effect on DPPH^•^ Radicals

The capacity to scavenge the free radical 2,2-diphenyl-1-picrylhydrazyl (DPPH^•^) was monitored with the method reported by Król et al. [31] with some modifications. The extract solution (10 µL of the extract diluted 1:5 in methanol) was mixed with 2 mL of a methanol solution containing DPPH^•^ radical (30 µL/mL). The mixture was shaken vigorously and left to stand for 30 min at room temperature in the dark (until absorbance values were stable).

Reduction of the DPPH^•^ radical was measured by monitoring the absorption decrease at 515 nm. The DPPH scavenging effect was calculated with the following equation:

% scavenging effect = [(ADPPH − AS)/ADPPH] × 100(1)

where A_DPPH_ is the absorbance of the DPPH^•^ solution and A_S_ is the absorbance of the DPPH^•^ solution after addition of the sample extract. The amount of matrix used to prepare an extract providing 50% inhibition (EC50) was extrapolated from the % scavenging effect. 

#### 3.6.2. Scavenging Effect on ABTS^+•^ Radicals 

The ABTS method was applied as per Król et al. [31] with slight modifications. The ABTS radical was generated by chemical reaction with potassium persulfate (K_2_S_2_O_8_). First, 5 mL of K_2_S_2_O_8_ solution (0.66 mg/mL) were added to 5 mL of ABTS (3.84 mg/mL), then, the solution was kept in the dark for 12 to 16 h, at −20 °C, to form the radical. An accurate volume of the previous solution was diluted in ethanol/water (50:50 *v*/*v*) until absorbance of 0.70 ± 0.02 at λ = 734 nm was achieved. Once the radical was formed, 2 mL of ABTS^+•^ radical solution was mixed with 100 μL of the extracts diluted 1:100 in ethanol, and the absorbance at λ = 734 nm measured after 6 min. The ABTS^+•^ scavenging effect was calculated as equivalent mmol of Trolox (6-hydroxy-2,5,7,8-tetramethylchroman-2-carboxylic acid) per g of matrix. The Trolox calibration curve was built up by analyzing the standard compound at concentrations ranging from 0.025 to 0.3 mM, with the same method.

#### 3.6.3. Offline Combination of Antioxidant Assays and HPLC-PDA Analysis

##### DPPH Method 

First, 50 μL of each extract were added to 10 mL of DPPH^•^ working solution, and left to stand for 30 min at room temperature in the dark; after solvent evaporation under a gentle nitrogen stream, the residue was diluted to 500 μL with methanol and filtered through a 0.22 μL PTFE filter. 5 μL of the resulting solution were submitted to LC analysis. The chromatographic pattern of the extract after reaction with DPPH^•^ radical solution was compared with that of the same extract before reaction, diluted 1:10.

##### ABTS Method 

First, 50 μL of extract were added to 10 mL of ABTS^+•^ working solution, and left to stand for 6 min at room temperature in the dark, then after evaporation under a gentle nitrogen stream the residue was diluted to a volume of 1 mL with methanol, and filtered through a 0.22 μL PTFE filter. 5 μL of the resulting solution were analyzed by LC. The chromatographic pattern of the extract after reaction with ABTS^+•^ radical solution was compared with that of the same extract before reaction, diluted 1:20.

For both the assays, the procedure was repeated thrice with highly consistent results (RSD% never exceeding 10%) and the percent peak area reduction was calculated on the mean areas.

### 3.7. Statistical Analysis

All analyses were performed in triplicate and data were expressed as mean values ± standard deviation. All statistical elaboration (experimental designs and their elaboration, principal component analysis, box plots, and Student’s *t*-test) were carried out using Statistica 10 (StatSoft. Inc., Tulsa, OK, USA) software. The experimental design was performed in order to optimize the extraction method of the raw plant material. Principal component analysis (PCA) was conducted to verify similarities and dissimilarities among the investigated samples, and box plots and Student’s *t*-test were used to define statistical differences concerning compound abundances and antioxidant power of the extracts (*p* < 0.01). 

## 4. Conclusions

This study reports the first phytochemical investigation on the polyphenolic pattern of *V. vinifera* green pruning residues (GPRs) by-products, generated by the annual pruning of vineyards in spring. The polyphenols were extracted under optimized experimental conditions, determined by experimental design, and identified and quantified, respectively, by HPLC-PDA-ESI-MS/MS and HPLC-PDA. Polyphenolics, principally consisting of flavonoids and phenolic acid derivatives, were the most interesting fraction. The phenolic phytochemical pattern of GPRs was compared with that of vine leaves and showed an equivalent composition with some differences in the ratio between components.

The antioxidant potential of GPR extracts was measured through colorimetric in vitro assays (scavenging of the 2,2-diphenyl-1-picrylhydrazyl (DPPH^•^) and the 2,2’-azinobis-3-ethyl-benzthiazoline-6-sulphonate (ABTS^+•^) radicals). The results were compared with those obtained for the leaf extracts and, then, with the results of HPLC investigation and total phenolic content assay, showing equivalent antioxidant properties for the two matrices and good consistency between the two techniques.

Taken together, the results suggest that grapevine GPRs are a potential source of natural compounds with valuable antioxidant properties that could be of interest in the pharmaceutical, chemical, and food fields, such as functional food ingredients. Valorization of these by-products could, in the future, have a major economic impact, because of their low cost and ready availability. At the same time, their re-use could also have a positive effect on the environment, as an aid to solving the problem of waste disposal associated with viticulture.

## Figures and Tables

**Table 1:** List of identified and putatively identified compounds in *V. vinifera* leaf and green pruning residue (GPR) extracts. For each analyte, retention time, UV maximum(a), pseudomolecular ions, and fragment ions obtained by product ion scan mode (PIS) and identified or tentatively identified compound names are given. Identification confidence values and references are also included.

N°	*t_R_* (min)	λ max (nm)	[M + H]^+^ m/z	[M − H]^−^ m/z	Mol. Weight (g/mol)	M^2+^ m/z	M^2−^ m/z	Aglycon (g/mol)	Compound Name ^†^	Leaves	GPRs	Identification Level ^§^	References
1	1.2	277	/	331	/	/	59,71,89,123,151,169,211	/	Galloylglucose	X	X	2	[39]
2	4.6	326/244	/	311	312	/	/	/	**Caftaric acid**	X	X	1	[27,40]
3	18.6	273	/	631	/	/	613, 479,445, 301, 273, 229	/	Hydrolyzable tannin	n.d.	X	3	[39,41,42]
4	19.6	SH 280	1431	1429	1430	321,303	753	/	Hydrolyzable tannin	n.d.	X	3	[41]
5	20.7	273	1431	1429	1430	1057,849,427,303	753	/	Hydrolyzable tannin	n.d.	X	3	[41]
6	22.1	SH 280	/	861,815,779	/	/	751, 301, 273	/	Hydrolyzable tannin	n.d.	X	3	[41,42]
7	22.9	348	495	493	494	319	317	318	Myricetin glucuronide	X	X	2	[23]
8	24.3	275	803	801	802	153, 337, 633	765	/	Vitilagin or isovitilagin	X	X	3	[43]
9	25.1	356	611	609	610	303	301	302	**Rutin**	X	X	1	[23,28,40]
		353	465	463	464	303	301	302	**Hyperoside**	X	X	1	[23,40]
10	25.5	254/352	465	463	464	303	301	302	**Quercetin 3-*O*-glucoside**	X	X	1	[23,40]
11	26	255/352	479	477	478	303	301	302	**Quercetin 3-*O*-glucuronide**	X	X	1	[23,40]
12	26.7	266/350	595	593	594	287	285	286	**Kaempferol 3-*O*-rutinoside**	X	X	1	
13	27.2	271/353	625	623	624	317	315	316	Isorhamnetin *O*-dihexoside (glucose+rhamnose)	X	X	2	
		271	479	477	478	317	315	316	Isorhamnetin hexoside	X	X	2	
14	27.5	264/349	449	447	448	287	285	286	**Kaempferol 3-*O*-glucoside**	X	X	1	[23,27,40]
		264/349	551	549	550	303	301	302	Quercetin malonylhexoside	X	X	2	
15	28.3	272/352	493	491	492	317	315	316	Isorhamnetin glucuronide	X	X	2	
16	29.5	275/353	535	533	534	287	285	286	Kaempferol malonylhexoside	X	X	2	
17	30.2	275	565	563	564	317	315	316	Isorhamnetin malonylhexoside	X	X	2	
		275	229	227	228	/	/	/	**Resveratrol**	traces	n.d.	1	[28]
18	36.4	368	303	301	302	/	/	/	**Quercetin**	X	X	1	[23,28]
19	37.5	368	317	315	316	/	/	/	**Isorhamnetin**	X	X	1	[23]
20	38.5	366	287	285	286	/	/	/	**Kaempferol**	X	X	1	[23,28]

^†^ In bold, the name of the compounds identified by comparison with authentic commercial reference standards. ^§^ Identification confidence as stipulated by the CAWG:46: Level 1, identified compound (a minimum of two independent and orthogonal data, such as retention time and mass spectrum) compared directly relative to an authentic commercial reference standard; Level 2, putatively annotated compound (compound identified by analysis of spectral data and/or similarity to data in a public database); and Level 3, putatively characterized compound class level.

**Table 2:** Percent peak area reduction of nine markers of green pruning residues (GPRs) and leaves after the reaction with the ABTS^+•^ and DPPH^•^ radicals (n.d. = not detected). For the compound number, see Table 1.

	Peak Reduction (%)			
	DPPH		ABTS	
**Compound N°**	**GPR**	**LEAVES**	**GPR**	**LEAVES**
**2**	38	33	10	31
**6**	33	n.d.	23	n.d.
**8**	72	77	64	84
**9**	30	30	31	42
**10**	33	29	30	41
**11**	44	21	29	40
**12**	<1	<1	8	36
**14**	<1	<1	6	12
**15**	<1	<1	27	17

## Supplementary Materials

The following are available online, Figure S1: Results of the optimization of extraction conditions obtained by experimental design relative to the sum of the peak areas; Figure S2: Results of the optimization of the extraction conditions obtained by the experimental design relative to the main components of the extracts; Figure S3: Box plots and histograms relative to the Folin–Ciocalteu assay; Figure S4: Box plots relative to the HPLC-PDA quantitative results on the main components of grapevine GPRs and leaves; Figure S5: Histograms relative to the in vitro colorimetric antioxidant assays for all GPRs and leaves extracts; Table S1: Variables, levels, and the design matrices evaluated in the two experimental design; Table S2: Pearson’s correlation matrix between HPLC-DAD quantitation results (in terms of sum of the concentrations of the main phenolics) and in vitro colorimetric antioxidant assays results; Table S3: List of the analyzed samples with their acronyms and sampling year; Table S4: Wavelengths, calibration ranges, equations of the curves and linearity of the target compounds used for quantification.
