# Supplementary material for: Grapevine Green Pruning Residues as a Promising and Sustainable Source of Bioactive Phenolic Compounds
Source: Molecules. 2020 Jan 22;25(3):464. doi: 10.3390/molecules25030464 (PMC7038121; doi:10.3390/molecules25030464)
Supplement: Supplementary file 1 [file molecules-25-00464-s001.pdf]

Supplementary materials

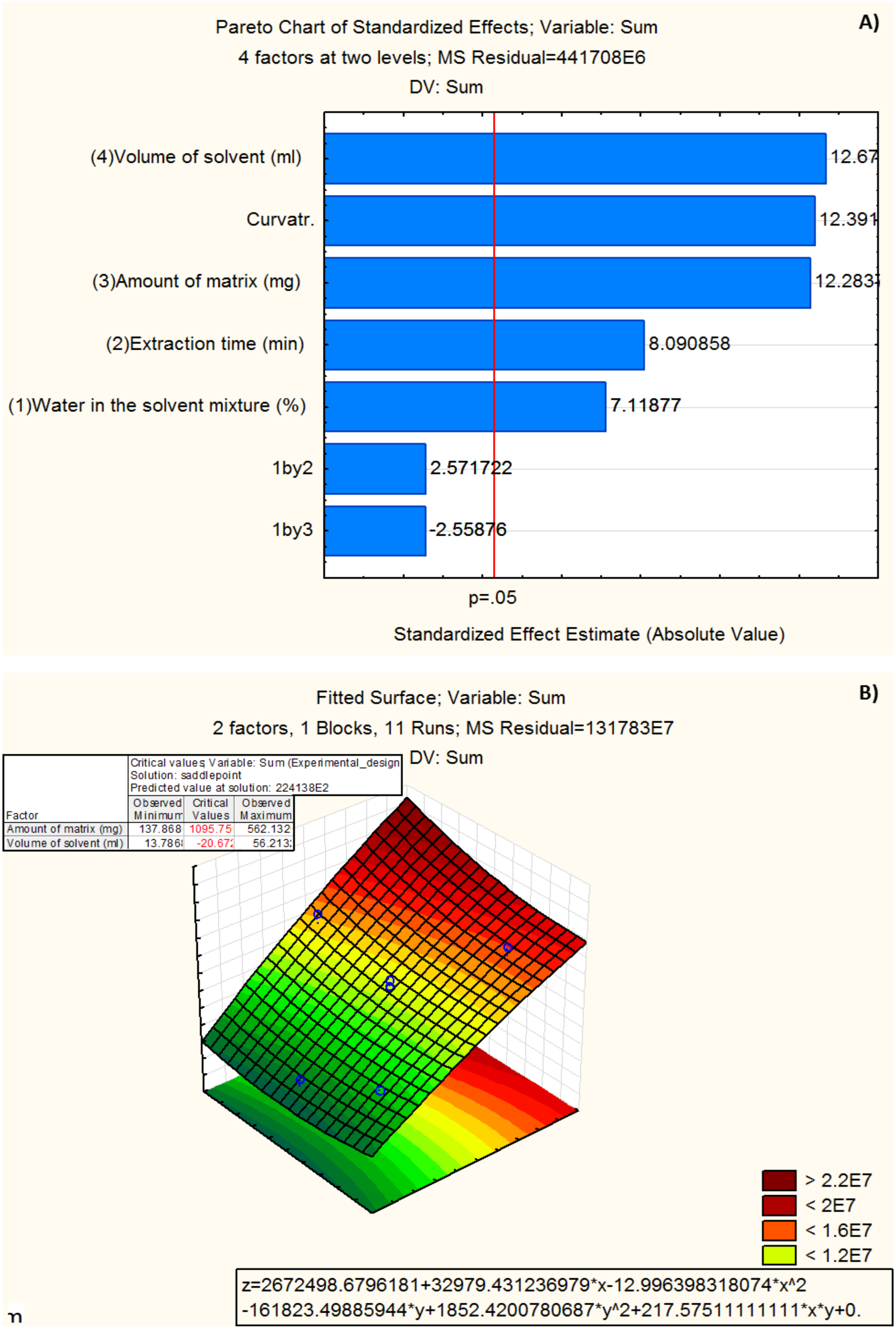

Figure S1. – Results of the optimization of extraction conditions obtained by experimental design: A) Pareto Chart of Effects relative to the sum of the peak areas of the HPLC-PDA chromatographic profiles of the extracts, obtained through the Box, Hunter & Hunter Design; B) Surface Response and

Critical Values relative to the sum of the peak areas of the HPLC-PDA chromatographic profiles of the extracts, obtained through the Central Composite Design.

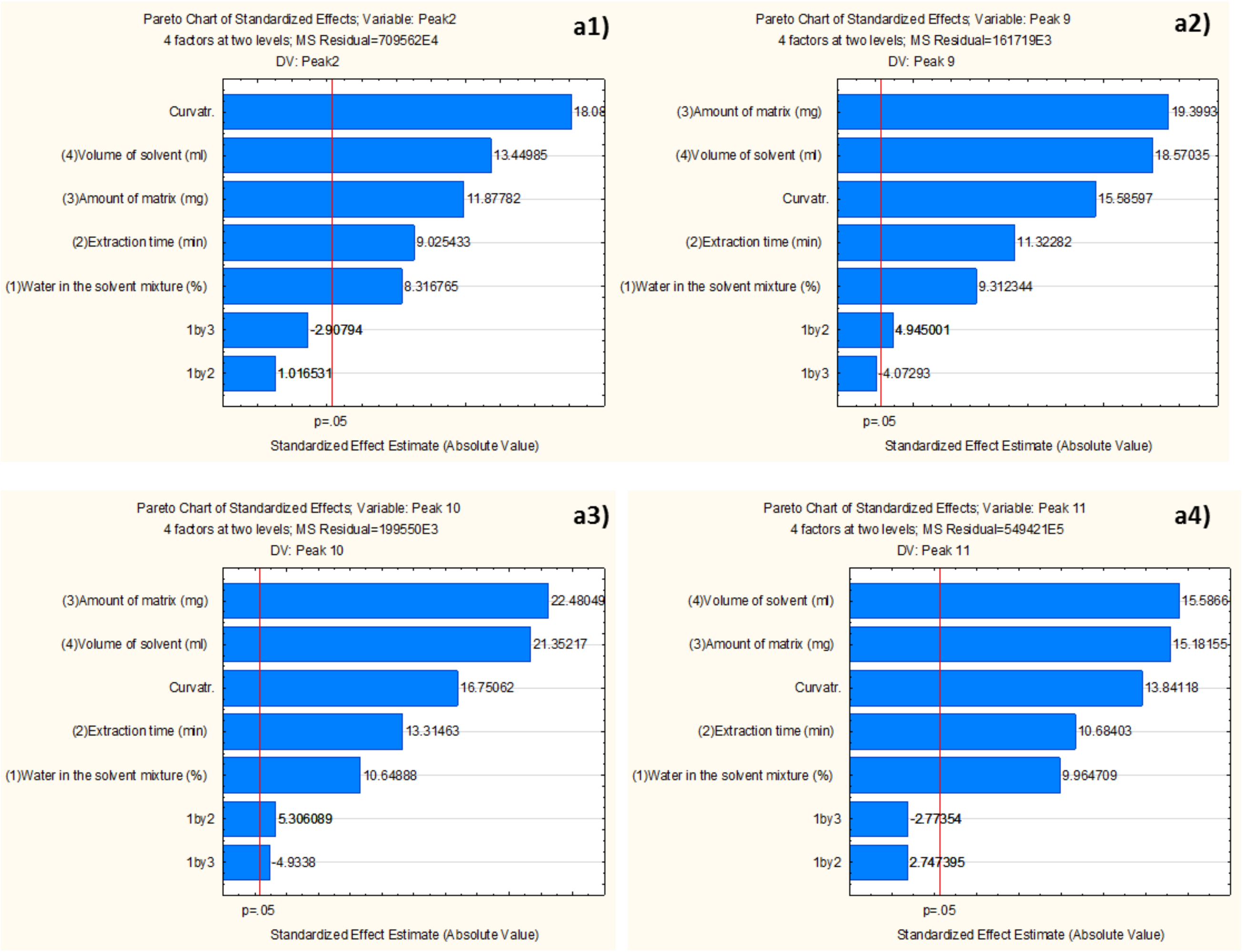

Pareto Chart of Standardized Effects; Variable: Peak2
4 factors at two levels; MS Residual=709562E4
DV: Peak2
a1)
Curvatr.
(4)Volume of solvent (ml)
(3)Amount of matrix (mg)
(2)Extraction time (min)
(1)Water in the solvent mixture (%)
1by3
1by2
18.08
13.44985
11.87782
9.025433
8.316765
-2.90794
1.016531
p=.05
Standardized Effect Estimate (Absolute Value)
Pareto Chart of Standardized Effects; Variable: Peak 9
4 factors at two levels; MS Residual=161719E3
DV: Peak 9
a2)
(3)Amount of matrix (mg)
(4)Volume of solvent (ml)
Curvatr.
(2)Extraction time (min)
(1)Water in the solvent mixture (%)
1by2
1by3
19.3993
18.57035
15.58597
11.32282
9.312344
4.945001
-4.07293
p=.05
Standardized Effect Estimate (Absolute Value)
Pareto Chart of Standardized Effects; Variable: Peak 10
4 factors at two levels; MS Residual=199550E3
DV: Peak 10
a3)
(3)Amount of matrix (mg)
(4)Volume of solvent (ml)
Curvatr.
(2)Extraction time (min)
(1)Water in the solvent mixture (%)
1by2
1by3
22.48049
21.35217
16.75062
13.31463
10.64888
5.306089
-4.9338
p=.05
Standardized Effect Estimate (Absolute Value)
Pareto Chart of Standardized Effects; Variable: Peak 11
4 factors at two levels; MS Residual=549421E5
DV: Peak 11
a4)
(4)Volume of solvent (ml)
(3)Amount of matrix (mg)
Curvatr.
(2)Extraction time (min)
(1)Water in the solvent mixture (%)
1by3
1by2
15.5866
15.18155
13.84118
10.68403
9.964709
-2.77354
2.747395
p=.05
Standardized Effect Estimate (Absolute Value)

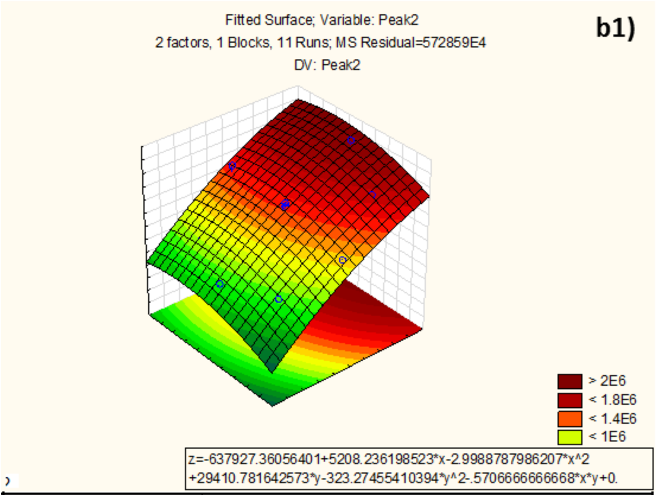

Factor	Critical values; Variable: Peak2 (Experimental_desigr<br>Solution: maximum<br>Predicted value at solution: 2270029.		
	Observed Minimum	Critical Values	Observed Maximum
Amount of matrix (mg)	137.868	864.108	562.132
Volume of solvent (ml)	13.786	44.726	56.213

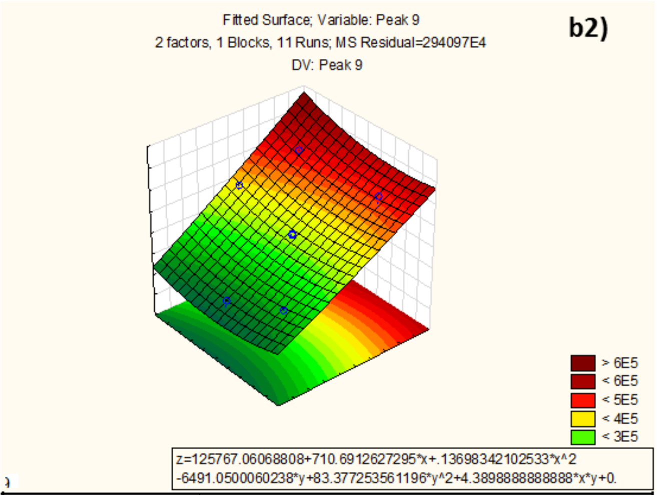

Factor	Critical values; Variable: Peak 9 (Experimental_desigr<br>Solution: minimum<br>Predicted value at solution: -24537E2		
	Observed Minimum	Critical Values	Observed Maximum
Amount of matrix (mg)	137.868	-5565.4	562.132
Volume of solvent (ml)	13.786	185.4	56.213

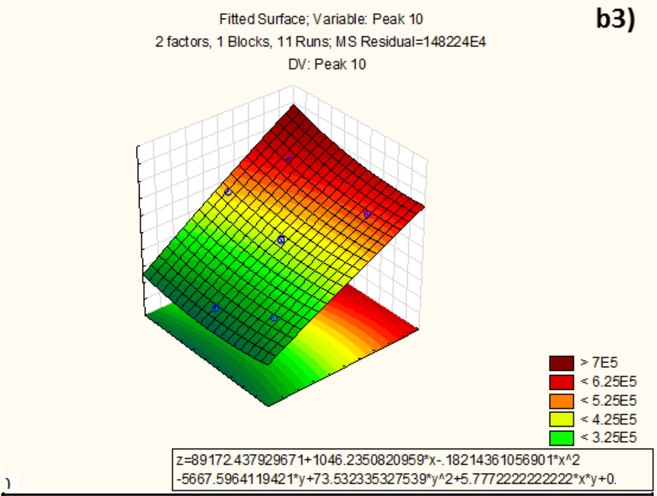

Factor	Critical values; Variable: Peak 10 (Experimental_desigr<br>Solution: saddlepoint<br>Predicted value at solution: 1341560.		
	Observed Minimum	Critical Values	Observed Maximum
Amount of matrix (mg)	137.868	2146.14	562.132
Volume of solvent (ml)	13.786	-45.77	56.213

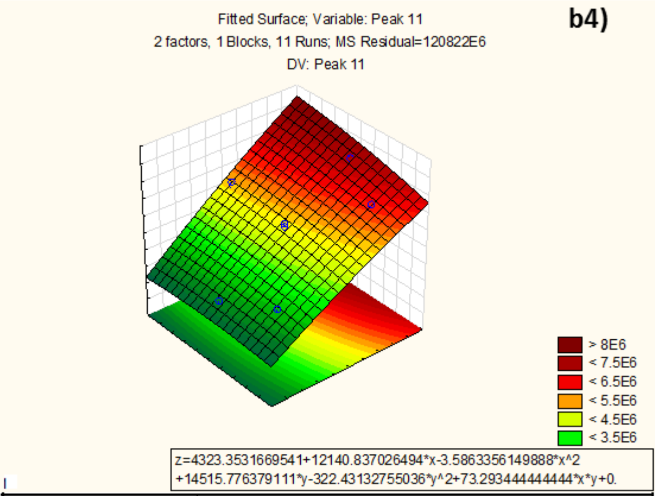

Factor	Critical values; Variable: Peak 11 (Experimental_desigr<br>Solution: saddlepoint<br>Predicted value at solution: -81972E3		
	Observed Minimum	Critical Values	Observed Maximum
Amount of matrix (mg)	137.868	-11912.4	562.132
Volume of solvent (ml)	13.786	-1331.4	56.213

Figure S2– Results of the optimization of the extraction conditions obtained by the experimental design. Pareto Chart of Effects relative to the main components of the extracts obtained through the Box, Hunter & Hunter Design: a1) peak 2, a2) peak9, a3) peak 10, a4) peak 11. Surface Response and Critical Values relative to the main components of the extracts obtained through the Central Composite Design: b1) peak 2, b2) peak9, b3) peak 10, b4) peak 11.

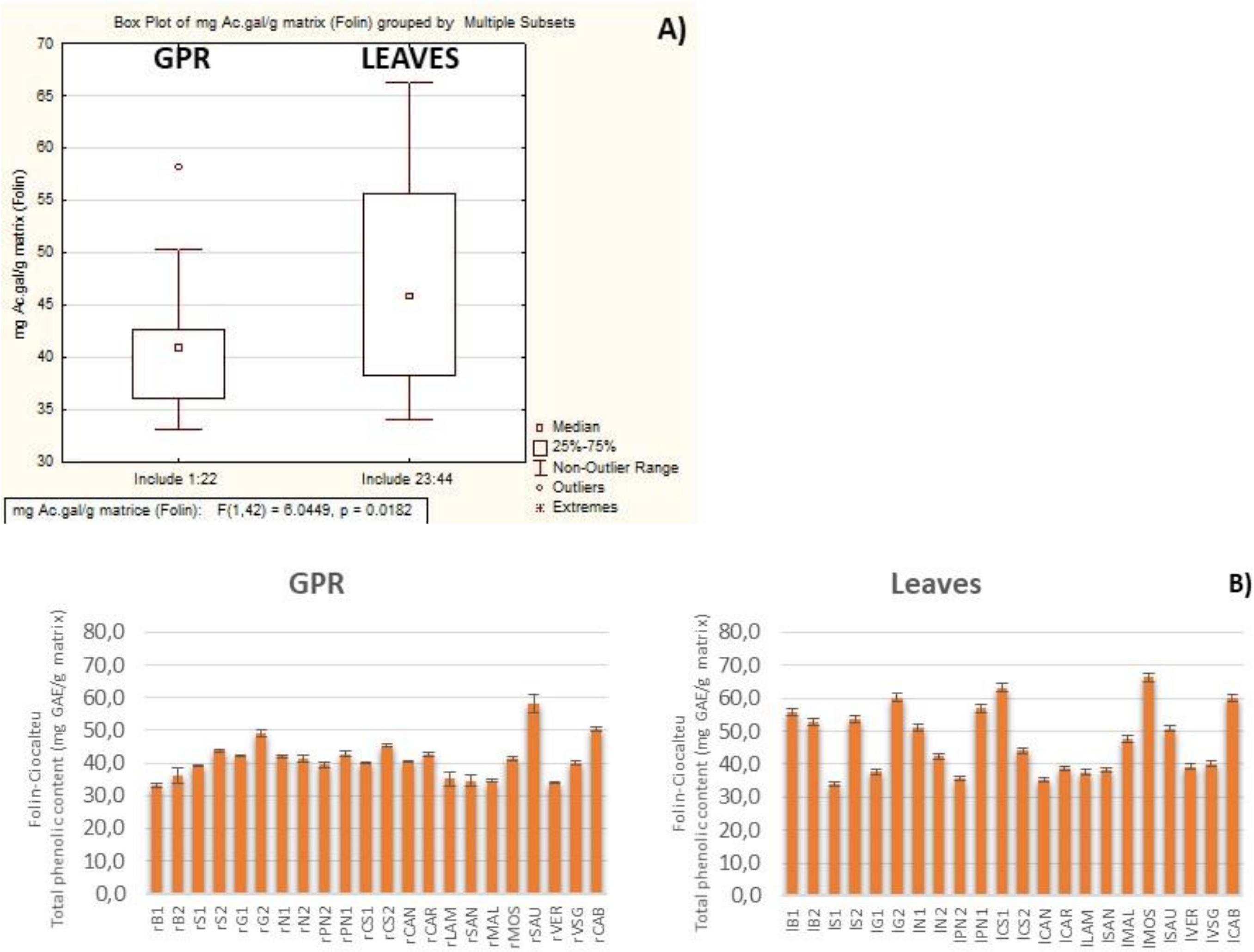

Figure S3. Box Plots (A) and histograms (B) relative to the Folin–Ciocalteu Assay. Total phenols content is expressed as mg GAE/g matrix for both the GPRs and leaves extracts.

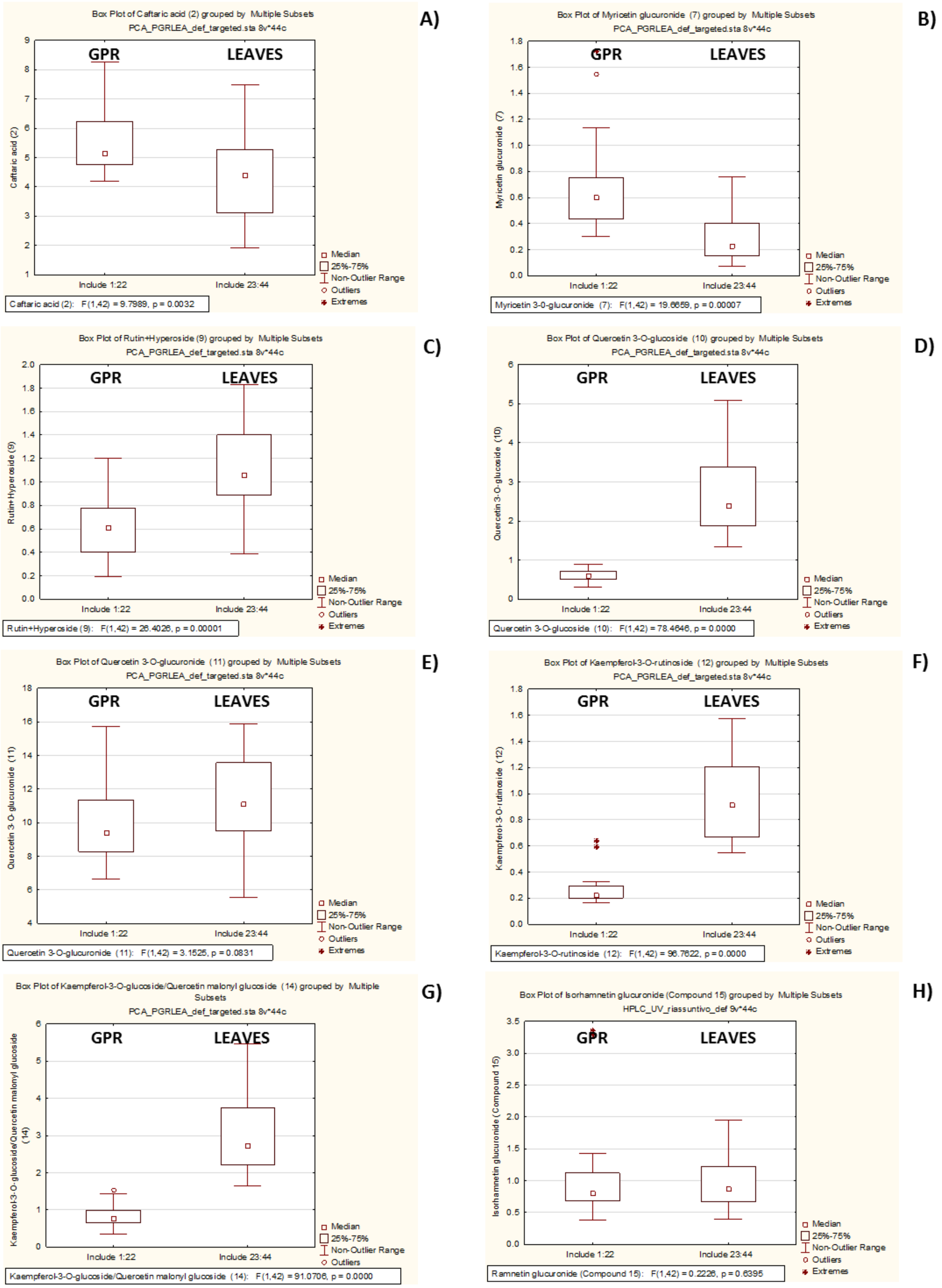

Figure S4. – Box Plots relative to the HPLC-PDA quantitative results on the main components of grapevine GPRs and leaves: A) caftaric acid, B) myricetin glucuronide, C) rutin and hyperoside, D) quercetin 3-*O*-glucoside, E) quercetin 3-*O*-glucuronide, F) kaempferol-3-*O*-rutinoside, G) kaempferol-3-*O*-glucoside and quercetin malonyl hexoside and H) isorhamnetin glucuronide.

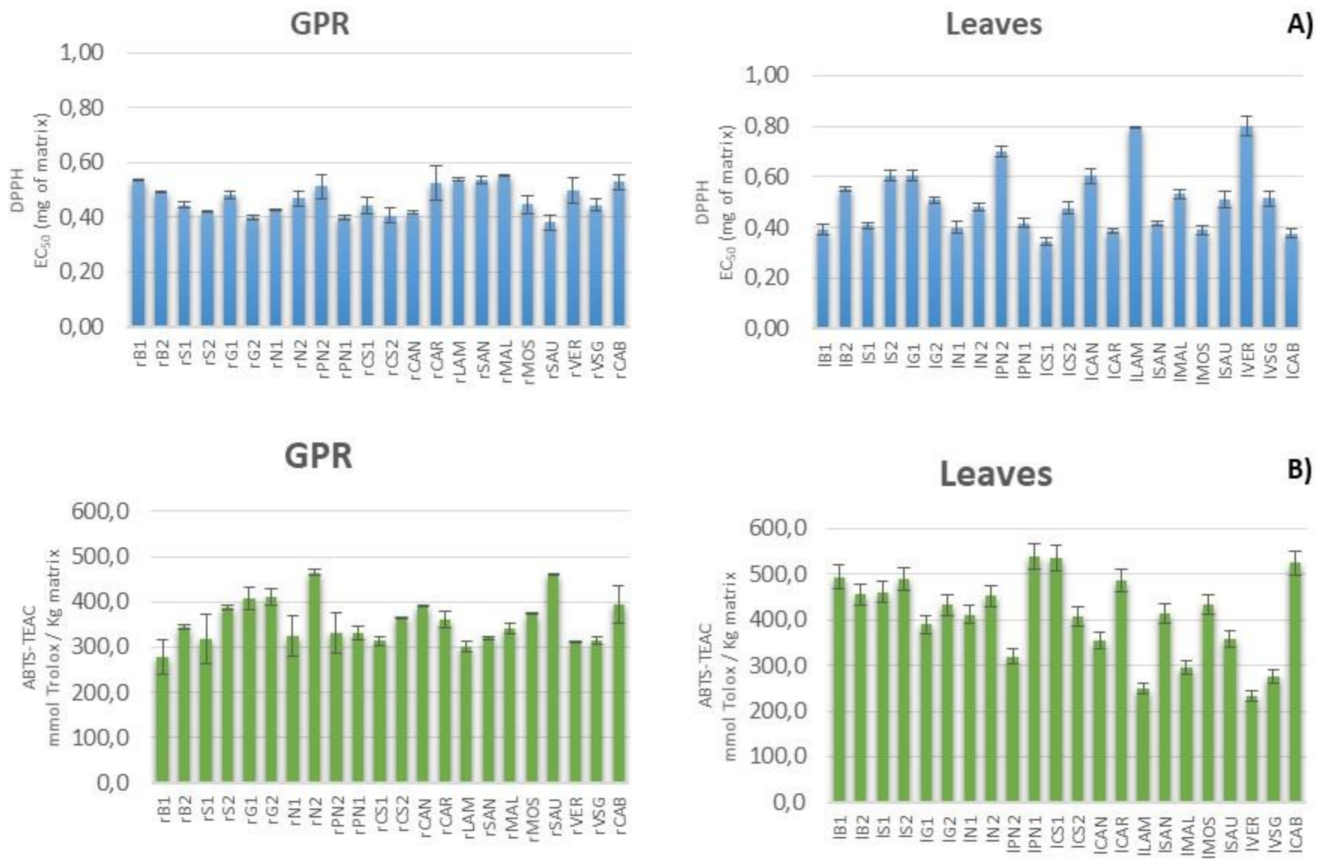

Figure S5. –Histograms relative to the *in vitro* colorimetric antioxidant assays for all GPRs and leaves extracts: EC_{50} (mg matrix) by DPPH Assay (A) and TEAC by ABTS Assay expressed as mmol Trolox/kg matrix (B).

Table S1. – Variables, levels and the design matrices evaluated in the two experimental design.

	INDEPENDENT VARIABLE	CODED VARIABLE	LEVELS				
			**-α**	**-1**	**0**	**+1**	**+α**
Box, Hunter & Hunter Design	Water in the solvent mixture (%)	*x1*		0	25	50	
	Extraction time (min)	*x2*		10	20	30	
	Amount of matrix (mg)	*x3*		100	250	500	
	Volume of solvent (ml)	*x4*		5	22.5	50	
Central Composite Design	Amount of matrix(mg)	*x3*	138	200	350	500	562
	Volume of solvent(ml)	*x4*	13.8	20	35	50	56.2

Box, Hunter & Hunter Design					Central Composite Design		
Run	*Variables*				Run	*Variables*	
	x1	*x2*	*x3*	*x4*		*x3*	*x4*
1	-1	-1	-1	-1	1	-1	-1
2	1	-1	-1	1	2	-1	1
3	-1	1	-1	1	3	1	-1
4	1	1	-1	-1	4	1	1
5	-1	-1	1	1	5	-α	0
6	1	-1	1	-1	6	+α	0
7	-1	1	1	-1	7	0	-α
8	1	1	1	1	8	0	+α
9	0	0	0	0	9	0	0
10	0	0	0	0	10	0	0
11	0	0	0	0	11	0	0

Table S2. – Pearson's correlation matrix between HPLC-DAD quantitation results (in terms of sum of the concentrations of the main phenolics) and *in-vitro* colorimetric antioxidant assays results.

Correlation matrix (Pearson (n-1))

Variable	Folin	ABTS	EC_{50} (DPPH)	HPLC
Folin	**1**	**0.6526**	**-0.4054**	**0.5612**
ABTS	**0.6526**	**1**	**-0.5701**	**0.6696**
EC_{50} (DPPH)	**-0.4054**	**-0.5701**	**1**	**-0.3257**
HPLC	**0.5612**	**0.6696**	**-0.3257**	**1**

Values in bold type differ from 0 at a significance level α=0.05

Table S3. –List of the analyzed samples with their acronyms and sampling year.

Cultivar	*Code*	*Sampling year*	*Cultivar*	*Code*	*Sampling year*
**Nebbiolo**	N1 – N2	2016	**Cabernet Franc**	CAB	2017
**Barbera**	B1 – B2	2016	**Canaiolo Nero**	CAN	2017
**Sirah**	S1 – S2	2016	**Carignano**	CAR	2017
**Grenache**	G1 – G2	2016	**Lambrusco Salamino**	LAM	2017
**Pinot Nero**	PN1 – PN2	2016	**Sangiovese**	SAN	2017
**Cabernet Sauvignon**	CS1 – CS2	2016	**Malvasia Bianca**	MAL	2017
**Moscato Bianco**	MOS	2017	**Verdicchio**	VER	2017
**Sauvignon Blanc**	SAU	2017	**Vernaccia**	VSG	2017

Table S4. Wavelenghts, calibration ranges, equations of the curves and linearity of the target compounds used for quantification.

Compound	*λ max (nm)*	*Linearity range (mg/l)*	R^2	*Calibration curve equation*
Caftaric acid	325	100-1000	0.9994	y=21869x-260461
Quercetin 3-O-glucuronide	350	100-1000	0.9992	y=14009x-235766
Rutin	350	5-250	0.9972	y=13724x-5740.1
Quercetin 3-O-glucoside	350	5-250	0.9979	y=16561x-9313.8
Kaempferol 3-O-glucoside	350	5-250	0.9979	y=3864x-3225.3
